# Mechano-Regulation of Trabecular Bone Adaptation Is Controlled by the Local *in vivo* Environment and Logarithmically Dependent on Loading Frequency

**DOI:** 10.3389/fbioe.2020.566346

**Published:** 2020-10-14

**Authors:** Ariane C. Scheuren, Paul Vallaster, Gisela A. Kuhn, Graeme R. Paul, Angad Malhotra, Yoshitaka Kameo, Ralph Müller

**Affiliations:** ^1^Institute for Biomechanics, ETH Zurich, Zurich, Switzerland; ^2^Institute for Frontier Life and Medical Sciences, Kyoto University, Kyoto, Japan

**Keywords:** bone adaptation, mechanical loading, *in vivo* micro-CT imaging, frequency dependency, micro-finite element analysis

## Abstract

It is well-established that cyclic, but not static, mechanical loading has anabolic effects on bone. However, the function describing the relationship between the loading frequency and the amount of bone adaptation remains unclear. Using a combined experimental and computational approach, this study aimed to investigate whether trabecular bone mechano-regulation is controlled by mechanical signals in the local *in vivo* environment and dependent on loading frequency. Specifically, by combining *in vivo* micro-computed tomography (micro-CT) imaging with micro-finite element (micro-FE) analysis, we monitored the changes in microstructural as well as the mechanical *in vivo* environment [strain energy density (SED) and SED gradient] of mouse caudal vertebrae over 4 weeks of either cyclic loading at varying frequencies of 2, 5, or 10 Hz, respectively, or static loading. Higher values of SED and SED gradient on the local tissue level led to an increased probability of trabecular bone formation and a decreased probability of trabecular bone resorption. In all loading groups, the SED gradient was superior in the determination of local bone formation and resorption events as compared to SED. Cyclic loading induced positive net (re)modeling rates when compared to sham and static loading, mainly due to an increase in mineralizing surface and a decrease in eroded surface. Consequently, bone volume fraction increased over time in 2, 5, and 10 Hz (+15%, +21% and +24%, *p* ≤ 0.0001), while static loading led to a decrease in bone volume fraction (−9%, *p* ≤ 0.001). Furthermore, regression analysis revealed a logarithmic relationship between loading frequency and the net change in bone volume fraction over the 4 week observation period (*R*^2^ = 0.74). In conclusion, these results suggest that trabecular bone adaptation is regulated by mechanical signals in the local *in vivo* environment and furthermore, that mechano-regulation is logarithmically dependent on loading frequency with frequencies below a certain threshold having catabolic effects, and those above anabolic effects. This study thereby provides valuable insights toward a better understanding of the mechanical signals influencing trabecular bone formation and resorption in the local *in vivo* environment.

## Introduction

It is well-established that cyclic, but not static loading has anabolic effects on bone (Hert et al., 1971; Lanyon and Rubin, 1984; Turner et al., 1995; Robling et al., 2001). This clear-cut discrepancy in osteogenic responses to both loading patterns highlights the key role of loading frequency in mechano-regulation of bone modeling and remodeling, collectively referred to as (re)modeling, the coordinated process by which bone is continuously formed and resorbed. Yet, the exact relationship between loading frequency and bone (re)modeling and bone adaptation remains unclear. While both experimental (Rubin and Mcleod, 1994; Turner et al., 1994; Hsieh and Turner, 2001) and theoretical studies (Turner, 1998; You et al., 2001) have suggested a dose-response relationship such that bone formation increases with higher loading frequencies, Warden and Turner have shown this relationship to be non-linear (Warden and Turner, 2004) using an axial loading model of mouse ulnae. Using this model, they showed that cortical bone adaptation increased with frequencies up to 5 and 10 Hz, but then plateaued thereafter. In line with these results, more recent *in silico* studies have found non-linear relationships between loading frequency and bone adaptation both in cortical (Tiwari and Kumar, 2018) as well as in trabecular (Kameo et al., 2011) bone. In the latter study, a single trabecula was subjected to cyclic uniaxial loading at frequencies of either 1, 3, 5, 10 or 20 Hz. Similar to the study by Warden et al., bone volume fraction increased up to 10 Hz but then plateaued thereafter (Kameo et al., 2011). However, owing to the lack of *in vivo* studies investigating the effects of loading frequency on trabecular bone adaptation, the validity of such *in silico* studies remains unclear. Furthermore, as frequency effects have been shown to vary depending on the anatomical region investigated (Zhang et al., 2007), the optimal frequency must be identified for every specific loading model.

Using a tail-loading model, we have previously shown that cyclic loading at a frequency of 10 Hz over 4 weeks elicits anabolic responses in mouse caudal vertebrae (Webster et al., 2008). Furthermore, by combining time-lapsed micro-computed tomography (micro-CT) imaging with micro-finite element (micro-FE) analysis, we were able to demonstrate that bone (re)modeling in the trabecular compartment is controlled by local mechanical signals at the tissue level (Schulte et al., 2013; Lambers et al., 2015; Webster et al., 2015). Specifically, by registering consecutive time-lapsed *in vivo* micro-CT images onto one another (Schulte et al., 2011), sites of bone formation and resorption were quantified in three dimensions and subsequently linked to corresponding mechanical signals calculated in the local *in vivo* environment (L*iv*E) (Schulte et al., 2013; Lambers et al., 2015; Webster et al., 2015). Herein, simulating the distribution of strain energy density (SED)—defined as the increase in energy associated with the tissue deformation per unit volume (i.e., a measure of direct cell strain) —within the caudal vertebrae revealed that bone formation was more likely to occur at sites of high SED, whereas bone resorption was more likely to occur at sites of low SED (Schulte et al., 2013; Lambers et al., 2015). While SED is widely used as a mathematical term to describe the mechanical signal influencing bone (re)modeling (Huiskes et al., 2000; Schulte et al., 2013; Birkhold et al., 2017; Cheong et al., 2020), other mechanical signals, such as interstitial fluid flow through the lacuna-canalicular network (LCN), are also known to play a major role in determining the local mechanical environment surrounding osteocytes, the main mechanosensors in bone (Fritton and Weinbaum, 2008; Weinbaum et al., 2011; Klein-Nulend et al., 2013). In this respect, it has been suggested that measures of fluid flow, such as the gradient in SED, would allow improved predictions of adaptive bone (re)modeling events (Webster et al., 2015; Tiwari et al., 2018). In this study, we therefore aimed to (1) investigate the effects of varying loading frequencies on the mechano-regulation of trabecular bone in mouse caudal vertebrae, (2) assess whether adaptive bone (re)modeling can be linked to mechanical signals in the local *in vivo* environment and (3) compare the modeling performance of SED and the gradient in SED for the prediction of local bone formation and resorption events on the tissue level. Specifically, we used time-lapsed *in vivo* micro-CT imaging to monitor bone adaptation over time in individual animals in response to cyclic loading at frequencies of 2, 5, and 10 Hz as well as in response to static loading. In comparison to conventional two-dimensional (2D) histomorphometric techniques, which have previously been used to investigate effects of varying frequencies on bone adaptation (Lanyon and Rubin, 1984; Hsieh and Turner, 2001; Robling et al., 2001; Zhang et al., 2007), the ability to quantify not only bone formation but also resorption over time could elucidate contrasting effects observed after static and cyclic loading. Furthermore, the analysis of various mechanical signals in the local *in vivo* environment by means of micro-FE analysis provided a better understanding of these signals influencing bone forming and resorbing cells on the local level. Finally, by determining the conditional probabilities for bone formation and resorption events to occur as a function of these mechanical signals (Schulte et al., 2013), this study contributed toward the description of the relationship between local mechanical signals and the subsequent mechano-regulation of bone adaptation. In future, these results will be highly beneficial for *in silico* studies aiming to predict the mechano-regulation of bone adaptation in response to various interventions.

## Results

### Trabecular Bone Adaptation to Load Is Dependent on Loading Frequency

In order to investigate the effects of varying loading frequencies on bone adaptation, we used an *in vivo* micro-CT approach (Lambers et al., 2011) to monitor bone adaptation of the sixth caudal vertebrae of C57BL/6J mice subjected to a 4-week loading regime of either sham (0 N), 8 N static or 8 N cyclic loading with frequencies of 2, 5, or 10 Hz, respectively. Table 1 shows the difference between the first and last time point (i.e., bone parameter_week4–week0_) of the bone structural parameters in the trabecular and cortical bone. In the trabecular bone compartment, the difference of bone volume fraction (BV/TV) and trabecular thickness (Tb.Th) between the first and last time point was significantly different between groups (*p* ≤ 0.0001), whereas no significant differences were detected between groups for the trabecular number and separation (Tb.N and Tb.Sp, *p* > 0.05). Whereas, the sham and static loading groups showed a net decrease in BV/TV and Tb.Th, the cyclic loading groups at 2, 5, and 10 Hz displayed increases in BV/TV and Tb.Th, with all of them being significantly different to the sham group (Table 1). With respect to the structural parameters of cortical bone, differences between the first and last time point were significantly different between groups for cortical area fraction (Ct.Ar/Tt.Ar, *p* ≤ 0.0001) and cortical thickness (Ct.Th, *p* ≤ 0.01), where the cyclic loading groups showed significantly greater increases compared to the sham-loaded group (Table 1).

**TABLE 1:** Difference between week 0 and week 4 for bone structural parameters in the trabecular and cortical compartments.

Morphometric parameter	Sham	Static	2 Hz	5 Hz	10 Hz	*p*-value
BV/TV (%)	−0.93 ± 0.789	−1.408 ± 1.392	2.333 ± 1.315****	3.240 ± 1.692****	3.680 ± 1.084****	≤0.0001
Tb.Th (mm)	0.006 ± 0.005	0.006 ± 0.005	0.021 ± 0.01***	0.020 ± 0.007**	0.021 ± 0.006***	≤0.0001
Tb.N (1/mm)	0.263 ± 0.122	−0.286 ± 0.076	−0.234 ± 0.127	−0.341 ± 0.082	−0.295 ± 0.221	>0.05
Tb.Sp (mm)	0.034 ± 0.019	0.037 ± 0.009	0.031 ± 0.024	0.043 ± 0.013	0.034 ± 0.027	>0.05
Ct.Ar/Tt.Ar (%)	0.507 ± 1.187	0.524 ± 1.931	2.746 ± 0.950*	3.838 ± 2.209**	3.496 ± 1.733**	≤0.0001
Ct.Th (mm)	0.004 ± 0.005	0.005 ± 0.005	0.013 ± 0.005*	0.014 ± 0.013*	0.014 ± 0.009*	≤0.01

P-values denote a significant difference between groups determined by one-way ANOVA, while “*” denotes significant difference to sham as assessed by multiple comparisons Dunnett’s test (*p < 0.05, **p ≤ 0.01, ***p ≤ 0.001, and ****p ≤ 0.0001).

**FIGURE 1:**
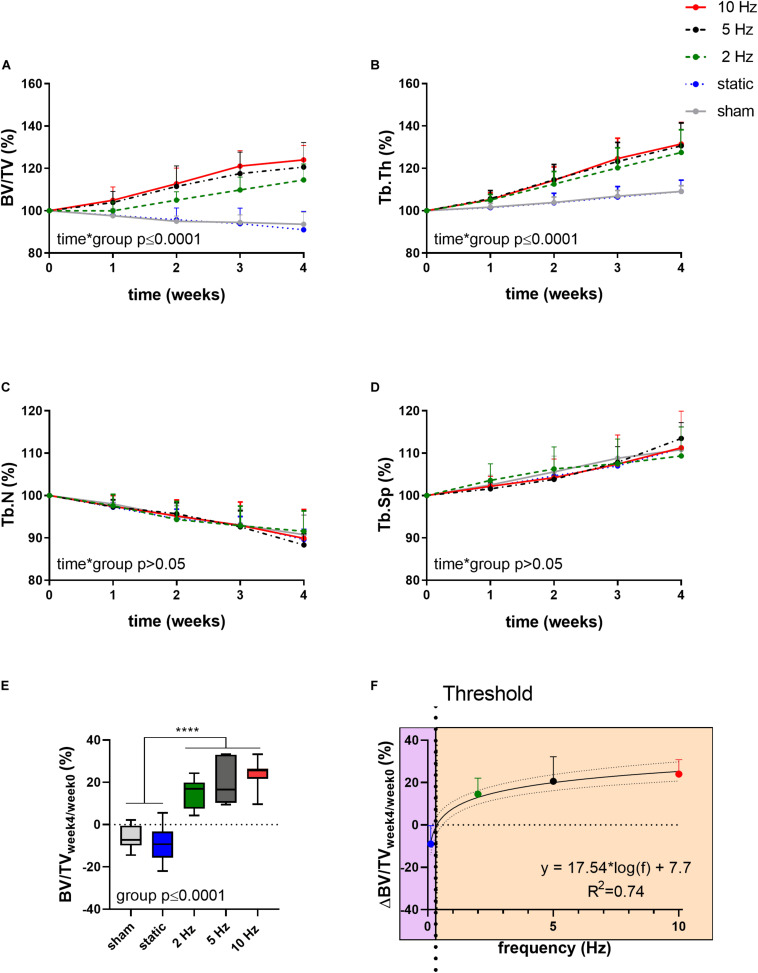
Relative changes of structural bone morphometric parameters in the trabecular compartment over the 4-week loading period as assessed by *in vivo* micro-CT. **(A)** Bone volume fraction (BV/TV), **(B)** trabecular thickness (Tb.Th), **(C)** trabecular number (Tb.N), and **(D)** trabecular spacing (Tb.Sp). (Data represent mean ± standard deviation (*SD*) for *n* = 5–8/group, *p*-values for interaction effect between group and time are shown as determined by linear mixed effects model). **(E)** The relative change from week 4 relative to baseline (BV/TV_week4/week0_) **(F)** was fitted with a logarithmic regression line. (Data represent mean ± *SD* for *n* = 5–8/group, *p*-value for main effect of group determined by one-way ANOVA, ****p ≤ 0.0001 denotes significant difference between groups determined by *post hoc* Tukey’s multiple comparisons test).

Figure 1 shows the relative changes in trabecular bone morphometric parameters over the 4-week loading period for the different loading groups. BV/TV developed differently over time between the loading groups (interaction effect, *p* ≤ 0.0001). Compared to the sham-loaded group, which showed no change in BV/TV over time (−6%, *p* > 0.05), cyclic loading at all frequencies (2, 5, and 10 Hz) led to a dose-response increase in BV/TV with higher frequencies resulting in higher increases in BV/TV (Figure 1A). Herein, the 5 and 10 Hz groups showed a significant increase compared to baseline already 2 weeks after the start of loading (*p* ≤ 0.001 and *p* ≤ 0.0001), while the 2 Hz group showed a significant increase relative to baseline only after 3 weeks (*p* ≤ 0.001). At the end of the 4-week loading regime, these groups showed a 15, 21, and 24% higher BV/TV relative to baseline (*p* ≤ 0.0001 for 2, 5, and 10 Hz). Static loading on the other hand, had catabolic effects resulting in significantly lower BV/TV (−9%, *p* ≤ 0.01) at the last time point relative to baseline. In line with the changes in BV/TV, Tb.Th developed differently over time between the loading groups (interaction effect, *p* ≤ 0.0001, Figure 1B). By the end of the 4-week loading intervention, all cyclic loading groups showed significant increases in Tb.Th (*p* ≤ 0.0001), which was not observed in the static and sham-loaded groups (*p* > 0.05). Although the number of trabeculae (Tb.N) decreased and trabecular separation increased (Tb.Sp) over time (Figures 1C,D, *p* ≤ 0.001), no relative differences were observed between the groups (*p* > 0.05). These results thus suggest that increases in BV/TV due to cyclic loading were mainly driven by thickening of the trabeculae rather than by the inhibition of the reduction in the number of trabeculae.

By plotting the relative changes in BV/TV as a function of loading frequency, regression analysis revealed a logarithmic relationship between bone adaptation and loading frequency as a best fit to the data (*R*^2^ = 0.74, Figure 1F) with loading frequencies above 0.36 Hz ± 0.08 having anabolic effects, and frequencies below this threshold having catabolic effects. Although there were no significant differences between the cyclic loading groups, loading at 10 Hz had the earliest and largest anabolic effects compared to the other frequencies.

**FIGURE 2:**
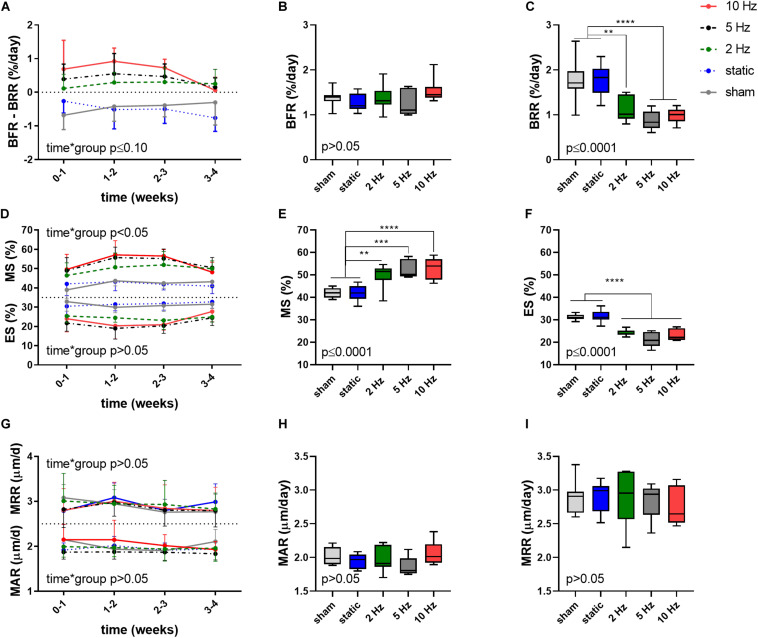
Dynamic bone morphometric parameters in the trabecular compartment in the different loading groups as assessed by *in vivo* micro-CT. **(A)** Changes in the net (re)modeling rate shown as the difference between bone formation rate (BFR) and bone resorption rate (BRR) over the 4-week loading period. Overall difference between groups of **(B)** BFR and **(C)** BRR. **(D)** Mineralized surface (MS) and eroded surface (ES) over the 4-week loading period. Overall difference between groups of **(E)** MS and **(F)** ES. **(G)** Mineral apposition rate (MAR) and mineral resorption rate (MRR) over the 4-week loading period. Overall difference between groups of **(H)** MAR and **(I)** MRR. [Data represent mean ± *SD* for *n* = 5–8/group, *p*-values for interaction effect between group and time are shown as determined by linear mixed effects model **(A,D,G)**, boxplots showing the differences between groups as determined by Tukey’s *post hoc* multiple comparisons test *p < 0.05, **p ≤ 0.01, ***p ≤ 0.001, ****p ≤ 0.0001 **(B,C,E,F,H,I)**].

Aside from providing information on changes in bone structural parameters over time, *in vivo* micro-CT also provided the possibility to assess dynamic bone formation and resorption activities such as bone formation/resorption rate (BFR/BRR), mineral apposition/resorption rate (MAR/MRR) and mineralizing/eroded surface (MS/ES) (Schulte et al., 2011). The net (re)modeling rate (BFR-BRR), which gives an indication whether there was overall bone gain (i.e., BFR-BRR>0) or loss (i.e., BFR-BRR<0) occurring within the trabecular compartment, tended to develop differently between groups (*p* ≤ 0.10). Compared to the static and sham-loaded groups, which had an overall negative (re)modeling balance, the 2, 5, and 10 Hz had an overall positive (re)modeling balance (*p* ≤ 0.01, *p* ≤ 0.001, and *p* ≤ 0.0001, Figure 2A). The net (re)modeling rate did not significantly change over time. When bone formation and resorption rates were analyzed separately, the main differences in the cyclic loading groups were in the reduced BRR as compared to the sham and static groups. While BFR did not significantly differ between groups (*p* > 0.05, Figure 2B), BRR was 35% (*p* ≤ 0.01), 50% (*p* ≤ 0.0001) and 44% (*p* ≤ 0.0001) lower in the 2, 5, 10 Hz groups, respectively, compared to the sham-loaded group (Figure 2C). The static group on the other hand had a similar BRR (−2%, *p* > 0.05) as the sham-loaded group (Figure 2C).

A difference between the cyclic and static loading groups was also apparent when investigating the surfaces of formation (mineralized surface, MS, interaction effect *p* < 0.05) and resorption (eroded surface, ES, interaction effect *p* > 0.05) sites with the cyclic loading groups having a higher MS and lower ES compared to the static and sham-loaded groups (Figure 2D). On average, formation sites occupied 2, 2.5, and 2.6 more surfaces than resorption sites for the 2, 5, and 10 Hz groups, and only 1.4 times more for the control and static groups, respectively.

Furthermore, the 2, 5, and 10 Hz groups had a 18% (*p* ≤ 0.01), 25% (*p* ≤ 0.001) and 26% (*p* ≤ 0.0001) higher mineralized surface (MS) and a 22% (*p* ≤ 0.0001), 32% (*p* ≤ 0.0001) and 26% (*p* ≤ 0.0001) lower eroded surface (ES) compared to the sham-loaded group, while the static group had similar MS and ES compared to sham-loading (*p* > 0.05, Figures 2E,F). The mineral apposition and resorption rates (MAR and MRR), which represent the thicknesses of formation and resorption packages, respectively, did not develop differently between groups (interaction effects *p* > 0.05). Furthermore, the MAR and MRR were similar between groups (*p* > 0.05), thus suggesting that they are not affected by loading (Figures 2G–I). This indicates that cyclic loading had a greater effect on surface than on thickness of formation as well as resorption sites.

### Trabecular Bone Adaptation to Load Is Controlled by Mechanical Signals in the Local *in vivo* Environment

**FIGURE 3:**
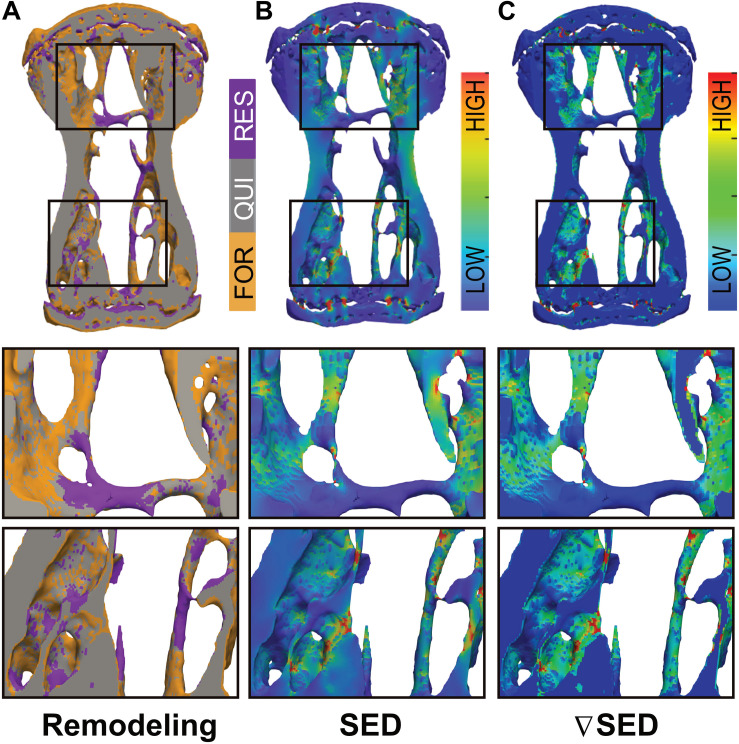
Qualitative visualization linking bone (re)modeling sites (formation, quiescence, resorption) with the mechanical environments *in vivo*. **(A)** Overlay of time-lapsed micro-CT images showing sites of bone formation (orange), quiescence (gray) and resorption (purple). Corresponding map of the **(B)** strain energy density (SED) and **(C)** gradient thereof (▽SED) showing sites of higher (red) and lower (blue) SED/▽SED values obtained by micro-finite element (micro-FE) analysis.

In order to assess whether bone (re)modeling events—namely formation, quiescence [i.e., where no (re)modeling occurred] and resorption—can be linked to the corresponding mechanical signals in the local *in vivo* environment, we performed micro-finite element (micro-FE) analysis to calculate the strain distribution within the tissue. As deformation (direct cell strain) and interstitial fluid flow (shear stress) are hypothesized to be the main mechanical stimuli that regulate load-induced bone adaptation (Rosa et al., 2015), we quantified the strain energy density (SED) magnitudes as a measure of mechanical deformation and the spatial gradient thereof (▽SED), as a measure of fluid flow (Kufahl and Saha, 1990; Webster et al., 2015). Figure 3 displays a representative visualization of a section of the vertebrae of the 10 Hz group showing sites of bone (re)modeling (Figure 3A) as well as the corresponding maps of SED (Figure 3B) and ▽SED (Figure 3C). From this qualitative analysis, it is apparent that bone resorption occurs at sites of lower SED and ▽SED, respectively, whereas bone formation occurs at sites of higher SED and ▽SED (Figure 3).

**FIGURE 4:**
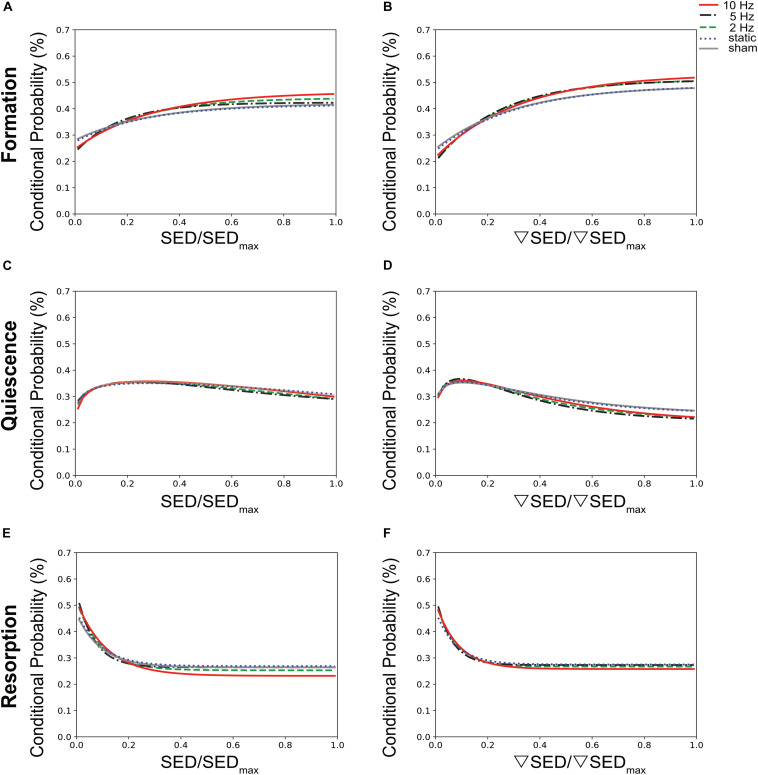
Conditional probabilities connecting SED (left side) and SED gradient (▽SED, right side) with (re)modeling events. The plots show the exponential fitting functions for **(A,B)** bone formation (top row), **(C,D)** quiescence (middle row), and **(E,F)** resorption (bottom row) in all the loading groups averaged over all time points.

To establish a quantitative description of the mechano-regulation of bone (re)modeling, we calculated the conditional probabilities for a given (re)modeling event to occur as a function of the mechanical stimuli, also known as (re)modeling rules (Schulte et al., 2013). Figure 4 shows the conditional probability curves for formation (orange), quiescence (gray) or resorption (purple) to occur at a given value of SED (Figures 4A,C,E) or ▽SED (Figures 4B,D,F) for the different groups averaged over all time points. For all groups, the conditional probability for bone formation to occur was higher at higher values of SED and ▽SED, respectively (SED/SED_max_ > 0.18) whereas bone resorption was more likely to occur at lower values (SED/SED_max_ < 0.18). The probability curves for all groups were fit by exponential functions (Supplementary Table S1), of which the coefficients provide information on the functioning of the mechanosensory system as described previously (Schulte et al., 2013). When comparing the slopes of the formation probability curves (parameter a, Figures 4A,B and Supplementary Table S1), which can be interpreted as the mechanical sensitivity of the system, there was a gradual increase of the mechanical sensitivity with increasing frequency with the 10 Hz group showing the highest mechanical sensitivity [a_(SED)_ = 0.217, a_(SEDgrad)_ = 0.316]. For the resorption probability curves (Figures 4E,F and Supplementary Table S1), the 5 and 10 Hz groups showed similar mechanical sensitivity to SED [a_(SED)_ = 0.284], while the 5 Hz group showed highest sensitivity to ▽SED [a_(SEDgrad)_ = 0.264 compared to a_(SEDgrad)_ = 0.252 in 10 Hz group]. The probability of the quiescence however, was not influenced by loading frequency (Figures 4C,D). The presence of an offset-parameter y_0_ in all loading groups indicates a certain probability for bone formation and resorption to occur over the full range of mechanical stimuli. These results thus suggest a baseline of bone (re)modeling, which is independent of mechanical stimuli, also referred to as non-targeted (re)modeling (Parfitt, 2002; Schulte et al., 2013). Compared to the sham and static loading groups, cyclic loading lowered the probability for non-targeted bone formation to occur as shown by lower y_0_ values in these groups (Figures 4A,B and Supplementary Table S1). Regarding the probability for non-targeted bone resorption to occur, only minor differences between loading groups were observed. The static loading group showed the highest y_0_ values [y_0(SED)_ = 0.269, y_0(SEDgrad)_ = 0.275, whereas the 10 Hz group showed the lowest values (y_0(SED)_ = 0.232, y_0(SEDgrad)_ = 0.258, Figures 4E,F and Supplementary Table S1]. When comparing between SED and ▽SED as mechanical stimuli driving bone (re)modeling events, it seems that in all groups, formation was more sensitive to ▽SED shown by the higher slopes [a_(SED)_ < a_(SEDgrad__)_] of the probability curves (Figures 4A,B and Supplementary Table S1). In contrast, resorption seemed to be more sensitive to SED [a_(SED)_ > a_(SEDgrad)_, Figures 4E,F and Supplementary Table S1].

**FIGURE 5:**
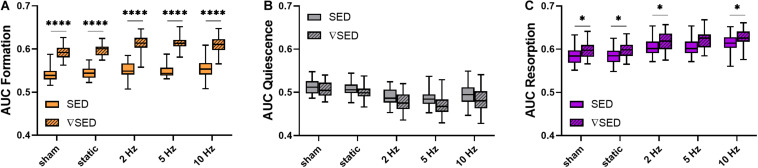
Area under the curve (AUC) values for the comparison of the modeling performance of SED and SED gradient. **(A)** Formation (orange), **(B)** quiescence (gray), and **(C)** resorption (purple) sites for the different loading groups comparing modeling performance of SED (solid bars) and SED gradient (▽SED, striped bars). (Boxplots for *n* = 5–8/group, ^∗^*p* < 0.05, ^****^p ≤ 0.0001 differences between groups determined by Tukey’s multiple comparisons test).

To better compare the modeling performance of SED vs. ▽SED for the prediction of bone (re)modeling events, an area under the receiver operator characteristic curve (AUC) approach was used (Figure 5). For all groups, the AUC values for formation (for all groups *p* ≤ 0.0001, Figure 5A) and resorption (for all groups *p* < 0.05 except for 5 Hz *p* ≤ 0.10, Figure 5C) events were higher for the ▽SED compared to SED. No difference between SED and ▽SED was observed for quiescence (Figure 5B). These results suggest that ▽SED has a better modeling performance compared to SED for determining the probability of bone formation and resorption events.

## Discussion

In this study, the effects of cyclic loading at varying frequencies as well as of static loading on trabecular bone adaptation in mouse caudal vertebrae were investigated. Furthermore, using a combination of *in vivo* micro-CT and micro-FE analysis, we assessed whether local bone (re)modeling events (formation and resorption) can be linked to diverse mechanical environments *in vivo*.

While static loading had catabolic effects, cyclic loading at 2, 5, and 10 Hz had anabolic effects on trabecular bone. In line with previous studies using the tail loading model (Lambers et al., 2011, 2015), cyclic loading over 4 weeks led to an increase in BV/TV, which was driven by the thickening of individual trabeculae rather than a prevention of loss in trabecular number. Furthermore, by registering consecutive time-lapsed images onto one-another, we were able to quantify both bone formation as well as bone resorption activities in three dimensions (Lambers et al., 2011), which to the best of our knowledge, has not yet been used to assess the effects of static loading regimes. Specifically, we showed that cyclic loading mainly affects the surfaces of the bone formation and resorption sites (MS and ES), rather than the thickness of these (re)modeling packets (MAR and MRR). In agreement with previous studies (Lambers et al., 2011; Schulte et al., 2011), these results suggest that cyclic loading promotes osteoblast recruitment, while simultaneously inhibiting osteoclast recruitment. Ultimately, cyclic loading results in larger mineralized surfaces and smaller eroded surfaces while keeping the thickness of the (re)modeling packets constant.

Notably, this study showed a logarithmic relationship between loading frequency and load-induced trabecular bone adaptation with frequencies above a certain threshold having anabolic effects and those below having catabolic effects. That cyclic, but not static loading, has anabolic effects on cortical bone has been shown in various animal models including rabbits (Hert et al., 1971), turkeys (Lanyon and Rubin, 1984) and rats (Turner et al., 1995; Robling et al., 2001). However, to the best of our knowledge, the effect of static loading has not yet been assessed in trabecular bone in mice. In line with the existence of a frequency threshold (0.36 Hz ± 0.08) to elicit anabolic responses as demonstrated in this study, Turner et al. (1994) found that bone formation rate in rat tibiae only increased with frequencies above 0.5 Hz, followed by a dose-response increase up to 2 Hz. Using a similar design as our study, Warden and Turner (2004) showed increased cortical bone adaptation with increasing loading frequencies up to 5–10 Hz with no additional benefits beyond 10 Hz. In a theoretical study, Kameo et al. (2011) furthermore showed similar results by subjecting individual trabeculae to uniaxial loading at frequencies ranging from 1 to 20 Hz. Although one would expect higher loading frequencies to lead to higher cellular stimulation and a consequent greater anabolic response, it has been suggested that frequencies above a certain threshold (10 Hz) reduce the efficiency of fluid flow through the LCN, thus resulting in inefficient mechanotransduction (Warden and Turner, 2004; Kameo et al., 2008). More recently, by monitoring Ca^2+^ signaling in living animals, Lewis et al. (2017) have shown that osteocyte recruitment was strongly influenced by loading frequency. Another physiological system, for which the relationship between frequency and mechanotransduction is widely studied, is the inner ear (Vollrath et al., 2007; Jaalouk and Lammerding, 2009). Hair cells, the cells responsible for transducing mechanical forces originating from acoustic waves to neural signals, are sensitive to frequency (Jaalouk and Lammerding, 2009; Salvi et al., 2015). Furthermore, the sensitivity of the ear varies with the frequency of sound waves resulting in a limited range of frequencies that can be perceived. Hence, drawing an analogy to the theory of sound pressure level, which also displays logarithmic laws (Beranek and Mellow, 2012), it is possible that bone’s response to frequency is similar to the perception of sound in human hearing.

Using the combined approach of time-lapsed *in vivo* micro-CT imaging and micro-FE analysis, we showed that bone (re)modeling activities were correlated to the local mechanical environment at the tissue level. In agreement with previous studies (Schulte et al., 2013; Lambers et al., 2015), bone formation was more likely to occur at sites of higher SED whereas bone resorption was more likely to occur at sites of lower SED. Nevertheless, as is also evident in the qualitative visualization in Figure 3, a one-to-one relationship between (re)modeling and the local mechanical environment cannot always be found. In this respect, we observed that in all loading groups, a certain amount of (re)modeling occurred independent of mechanical stimuli [i.e., non-targeted (re)modeling], in agreement with previous studies (Schulte et al., 2013; Lambers et al., 2015). Indeed, it is well-established that the regulation of bone (re)modeling is not limited to mechanical stimuli, and that various other factors such as systemic hormones [e.g., parathyroid hormone (PTH), estrogen] and growth factor signals (e.g., IGFs, BMPs) need to be considered (Siddiqui and Partridge, 2016). While our analysis does not allow to distinguish between mechanical and other stimuli as the major factor influencing one single (re)modeling site, we were able to show that the probability for such an event to occur changes with the mechanical signal. Specifically, compared to static loading, cyclic loading decreased the probability of non-targeted bone (re)modeling, which led to an increase in bone formation and a decrease in bone resorption. As the amount of non-targeted bone (re)modeling is expected to be the same in all mice, these results suggest that cyclic loading increases the amount of mechanically driven (re)modeling, thus leading to the clearer SED and SED gradient dependency. Furthermore, considering the number of (re)modeling sites per animal as well as the number of mice within the different loading groups, we believe that the quantification of the probabilities for certain (re)modeling events to occur at a given mechanical signal represents a suitable method to investigate the relationship between the (re)modeling and the local mechanical environments.

By comparing various mechanical stimuli as drivers for bone (re)modeling, we showed that the SED gradient was better at predicting bone formation and resorption events compared to SED. That the SED gradient, a measure of fluid flow through the LCN, can improve predictions of (re)modeling events compared to SED, a measure of direct cell strain, has been suggested previously (Webster et al., 2015). Furthermore, as the SED gradient encompasses the neighboring SED voxels, it provides information of a broader mechanical environment, which could explain the higher modeling performance observed with the SED gradient compared to SED.

There are a number of limitations to consider in this study. Firstly, as the strain magnitude and duration of individual loading bouts were the same for all loading groups, the number of cycles and strain rate differed between the different loading groups. From this study design, it therefore remains impossible to know whether the number of cycles or the loading frequency are the main factors driving load-induced bone adaptation. Furthermore, loading at low (1 Hz) and higher (>10 Hz) frequencies was not assessed in this study. Indeed, mechanical stimulation at very high frequencies (between 20 and 90 Hz), but at low magnitude, also referred to as “low magnitude high frequency vibration (LMHFV)” has been shown to elicit beneficial effects in bone during growth (Xie et al., 2006), disuse (Ozcivici et al., 2007), aging (Judex et al., 2007), and/or regeneration (Goodship et al., 2009; Gómez-Benito et al., 2011; Wehrle et al., 2014; Zhang et al., 2020). Herein, studies in ovariectomized rats (Judex et al., 2007) as well as in fractured sheep tibiae (Gómez-Benito et al., 2011) have shown frequency dependent effects with higher frequencies (90 Hz) being more beneficial compared to moderate frequencies (45–50 Hz). More recently however, Wehrle et al. (2014) showed anabolic effects on trabecular mouse bone in response to 35 Hz, whereas no effects were observed at 45 Hz. Interestingly, when the same LMHFV protocol was applied in fractured bones, no effects were observed at 35 Hz, whereas 45 Hz impaired the healing process. It therefore seems that frequency dependent effects of LMHFV differ not only between species but also between fractured and non-fractured bones. However, given the relatively high force magnitude (8 N) used in our cyclic loading protocol, the results obtained in this study cannot be directly compared with those observed with LMHFV applications. From a technical point of view, loading at much higher frequencies while maintaining such high forces would not be possible in our experimental set-up as the energy that is put into the system would be very high. Additionally, our results suggest that loading at higher frequencies would not be beneficial, as the system seems to be self-limiting at around 10 Hz, which has previously also been shown in a similar model of ulna loading (Warden and Turner, 2004). Nevertheless, whether bone’s osteogenic response to loading is indeed limited to a specific range of frequencies, below and above which bone becomes less osteogenic, requires further *in vivo* experiments.

A further limitation of this study is that the micro-FE analysis used to simulate the local mechanical environments was based on several assumptions. Specifically, the bone tissue was assumed to be an isotropic, homogenous material with all the bone elements having the same Young’s modulus (14.8 GPa) and Poisson’s ratio (0.3). Hence, the different degrees of mineralization within trabecular bone were not taken into account, which could influence the results observed in this study. This limitation could be overcome by including the evaluation of the dynamic mechanical properties of the bone samples by means of nanoindentation (Ozcivici et al., 2008). Alternatively, micro-FE models from gray-scale micro-CT images would allow to assign different values of Young’s moduli to voxels with different gray values, which could provide insight on the effects of loading frequency on mineralization both on a global and local level. In order to improve the micro-FE prediction of mechanical stimuli in a bone healing environment, Tourolle né Betts et al. (2020) recently developed a “multi-density threshold approach” to identify and quantify the spatial and temporal changes in local mineralization during fracture healing. Though a much smaller range of densities is expected in a model of adaptation compared to a model of regeneration, the integration of a multi-density approach could allow comparisons of variations of local mineralization in response to varying loading frequencies.

An additional major limitation of this study was that the micro-FE analysis did not take into account the component of frequency. Previous numerical studies have used poro-elastic finite-element (FE) models to account for lacunar-canalicular fluid motion and ultimately predict bone adaptation in response to varying loading frequencies (Kameo et al., 2011; Malachanne et al., 2011; Chennimalai Kumar et al., 2012; Pereira and Shefelbine, 2014). More recent applications of poro-elastic FE models have furthermore shown load-induced fluid velocity as an accurate predictor of local (re)modeling activities in mouse tibiae (Pereira et al., 2015; Carriero et al., 2018). In the later study, a combination of novel high-resolution techniques—known as “3D fluorochrome mapping” was used to link bone (re)modeling activities in the cortical bone both to strains (strain energy density) as well as to fluid flow velocities engendered by tibial loading. While high SED was able to predict periosteal bone formation, high fluid flow was able to predict bone formation on both the endosteal and periosteal surface. Hence, the incorporation of cellular mechanosensing and intercellular communication within our micro-FE models would be highly useful to improve our understanding of the relationship between loading frequency and trabecular bone adaptation across multiple scales.

Lastly, as cyclic loading has been shown to induce microscopic tissue damage (micro-damage) in human (Green et al., 2011; Lambers et al., 2013; Goff et al., 2015) and bovine (Lee et al., 2000; Moore and Gibson, 2004; Thurner et al., 2006) trabecular bone, it is possible that cyclic loading at varying frequencies results in different degrees of tissue micro-damage. We have previously imaged mouse caudal vertebrae subjected to cyclic loading at 10 Hz using high-resolution micro-CT (1.2 μm) and could not detect any micro-damage at the local level, and therefore, we do not expect the lower frequencies to cause local micro-damage either. Nevertheless, the incorporation of techniques to visualize bone micro-damage—ranging from 2D histology and 3D micro-CT imaging (Poundarik and Vashishth, 2015) to more advanced methods such as an automated step-wise micro-compression device for dynamic image-guided failure (Levchuk et al., 2018) —could provide novel insight into the initiation and propagation of micro-damage in response to cyclic loading in trabecular mouse bone.

In conclusion, these results suggest that bone adaptation is regulated by mechanical signals in the local *in vivo* environment and furthermore, that mechano-regulation is logarithmically dependent on loading frequency with frequencies below a certain threshold having catabolic effects, and those above anabolic effects. This study thereby provides valuable insights toward a better understanding of the mechanical signals influencing bone formation and resorption in the local *in vivo* environment.

## Materials and Methods

### Study Design

To investigate the effect of loading frequency on mouse caudal vertebrae, 11-week old female C57BL/6J mice were purchased (Charles River Laboratories, France) and housed at the ETH Phenomics Center (12 h:12 h light-dark cycle, maintenance feed and water ad libitum, three to five animals/cage) for 1 week. To enable mechanical loading of the 6th caudal vertebrae (CV6), stainless steel pins (Fine Science Tools, Heidelberg, Germany) were inserted into the fifth and seventh caudal vertebrae of all mice at 12 weeks of age. After 3 weeks of recovery, the mice received either sham (0 N), 8 N static or 8 N cyclic loading with frequencies of 2, 5, or 10 Hz and were scanned weekly using *in vivo* micro-CT. All procedures were performed under isoflurane anesthesia (induction/maintenance: 5%/1–2% isoflurane/oxygen). All mouse experiments described in the present study were carried out in strict accordance with the recommendations and regulations in the Animal Welfare Ordinance (TSchV 455.1) of the Swiss Federal Food Safety and Veterinary Office (license number 262/2016).

### Mechanical Loading

The loading regime was performed for 5 min, three times per week over 4 weeks as described previously (Webster et al., 2008). For the cyclic loading groups, sinusoidally varying forces (8 N amplitude) were applied at 2, 5 or 10 Hz resulting in cycle numbers of 600, 1,500, and 3,000, respectively. For the static loading group, the force was maintained at 8 N during the 5 min. For the sham-loaded group, the tails were fixed in the loading device for 5 min, but no loading was applied (0 N).

### Micro-CT Imaging and Analysis

*In vivo* micro-CT (vivaCT 40, Scanco Medical AG, isotropic nominal resolution: 10.5 μm; 55 kVp, 145 μA, 350 ms integration time, 500 projections per 180°, scan duration ca. 15 min, radiation dose per scan ca. 640 mGy) images of the CV6 were acquired every week. Micro-CT data was processed and standard bone microstructural parameters were calculated in trabecular, cortical and whole bone by using automatically selected masks for these regions as described previously (Lambers et al., 2011). To calculate dynamic morphometric parameters, micro-CT images from consecutive time-points were registered onto one another. The voxels present only at the initial time point were considered resorbed whereas voxels present only at the later time point were considered formed. Voxels that were present at both time points were considered as quiescent bone. By overlaying the images, morphometrical analysis of bone formation and resorption sites within the trabecular region allowed calculations of bone formation rate (BFR), bone resorption rate (BRR), mineral apposition rate (MAR), mineral resorption rate (MRR), mineralizing surface (MS), and eroded surface (ES) (Schulte et al., 2011).

### Micro-Finite Element (micro-FE) Analysis

For each mouse at each time point, segmented image data was converted to 3D micro-FE models, with additional voxels added to the proximal and distal ends of the vertebrae mimicking intervertebral disks. All voxels were converted to 8 node hexahedral elements and assigned a Young’s modulus of 14.8 GPa and a Poisson’s ratio of 0.3 (Webster et al., 2008). The bone was assumed to have linear elastic behavior, which allowed for static loading in the micro-FE analysis (Huiskes, 2000). The top was displaced by 1% of the length in z-direction (longitudinal axis), while the bottom was constrained in all directions. The micro-FE model was solved using a micro-FE solver (ParOSol). The results were then rescaled to an applied force of 8 N for the loaded groups and 4 N (physiological loading) for the sham-loaded group (0 N) as described previously (Christen et al., 2012).

### Mechanical Environment

The mechanical stimuli, which are hypothesized to drive load induced bone adaptation are deformation (direct cell strain) and interstitial fluid flow (shear stress) (Rosa et al., 2015). Furthermore, the flow velocity surrounding osteocytes has been shown to be dominant in the neighborhood of the bone surfaces (Kameo et al., 2008). As a measure of the mechanical deformation, strain energy density (SED) magnitudes, defined as the increase in energy associated with the tissue deformation per unit volume, were analyzed on the bone surface on the marrow-bone interface. Furthermore, based on the assumption that spatial differences in tissue deformation induce fluid flow, the spatial gradient of the SED was analyzed on the marrow side of the marrow-bone interface (Kufahl and Saha, 1990). The spatial gradients in x, y, and z-direction were calculated as follows:

$$\frac{\partial f_{i}}{\partial x,y,z}=\frac{f_{i+1}-f_{i-1}}{2a}\;\mathrm{for}\mathrm{voxel}1<i<N_{x}$$

Where *f*_*i*_ is the SED of a voxel at x, y, z-position *i*, *N*_*x,y,z*_ the number of voxels in the x, y, z-direction and *a* the nominal resolution. The norm of the gradient vector (▽SED) was used as a quantity for the fluid flow as described previously (Webster et al., 2015).

$$▽\text{SED}=\sqrt{{\left(\frac{\partial f_{i}}{\partial x}\right)}^{2}+{\left(\frac{\partial f_{i}}{\partial y}\right)}^{2}+{\left(\frac{\partial f_{i}}{\partial z}\right)}^{2}}$$

The conditional probabilities for a certain (re)modeling event (formation, quiescence, resorption) to occur at a given value of SED and ▽SED were calculated as described previously (Schulte et al., 2013). Briefly, the surface SED and ▽SED values were normalized within each animal and measurement by the maximal SED or ▽SED (chosen as the 99th percentile of the values present at the surface and in the volume of interest (VOI)) in order to remove the variance due to temporal bone adaptation, applied force in FE analysis and individual animals. For each region (formation, quiescence and resorption), a frequency density histogram with 50 bins and equal bin width was created. In order to rule out the dependence on the imbalance between bone formation and resorption, all (re)modeling events were assumed to have the same occurrence probability (i.e., formation, resorption and quiescent regions were rescaled to have the same amount of voxels). The (re)modeling probabilities were fitted by exponential functions using non-linear regression analysis.

To quantify the modeling performance of SED and ▽SED, respectively, the area under the curve (AUC) of a receiver operating characteristic (ROC) curve was used. The AUC can be defined as the probability that a randomly selected case (“true”) will have a higher test result than a randomly selected control (“false”) (Mason and Graham, 2002). The ROC curve is a binary classifier, therefore the three different surface regions were analyzed separately and only voxels and mechanical quantity values on the bone or marrow surface were used for the classification.

### Statistical Analysis

Data are represented as mean ± standard deviation (*SD*). For analysis of the longitudinal measurements of bone structural parameters, repeated measurements ANOVA implemented as a linear mixed model was used using the lmerTEST package (Kuznetsova et al., 2017) in R [R Core Team (2019), R Foundation for Statistical Computing, Vienna, Austria]. The between subjects effect was allocated to the different groups (sham, static, 2, 5, 10 Hz) while the within-subjects effects were allocated to time and time-group interactions. Random effects were allocated to the animal to account for the natural differences in bone morphometry in different mice. In cases where a significant interaction effect (group^∗^time) was found, a Tukey post-hoc multiple comparisons test was performed. For comparisons between groups one-way ANOVA analysis followed by Tukey’s or Dunnet’s multiple comparisons test were performed as stated in the corresponding figure legends using SPSS (IBM Corp. Released 2016. IBM SPSS Statistics for Windows, Version 24.0. Armonk, NY, United States). The plots were created using GraphPad Software (GraphPad Prism version 8.2.0 for Windows, GraphPad Software, La Jolla California, United States). Significance was set at α< 0.05 in all experiments.

## Data Availability Statement

All datasets presented in this study are included in the article/Supplementary Material.

## Ethics Statement

The animal study was reviewed and approved by the Animal Welfare Ordinance (TSchV 455.1) of the Swiss Federal Food Safety and Veterinary Office (license number 262/2016).

## Author Contributions

AS, GK, YK, and RM conceived and planned the experiments. AS, GK, and AM carried out the experimental experiments. PV carried out the computational experiments with the help of GP and AS. AS wrote the manuscript with the help of RM. All authors discussed the results, reviewed and commented on the manuscript.

## Conflict of Interest

The authors declare that the research was conducted in the absence of any commercial or financial relationships that could be construed as a potential conflict of interest.
